# Phase I trial of the oral PARP inhibitor olaparib in combination with paclitaxel for first- or second-line treatment of patients with metastatic triple-negative breast cancer

**DOI:** 10.1186/bcr3484

**Published:** 2013-09-25

**Authors:** Rebecca A Dent, Geoffrey J Lindeman, Mark Clemons, Hans Wildiers, Arlene Chan, Nicole J McCarthy, Christian F Singer, Elizabeth S Lowe, Claire L Watkins, James Carmichael

**Affiliations:** 1 National Cancer Center Singapore, Duke NUS, Singapore 169610, Singapore; 2 Sunnybrook Odette Cancer Center, Toronto, ON, Canada; 3 The Royal Melbourne Hospital & Walter and Eliza Hall Institute of Medical Research & University of Melbourne, Melbourne, VIC, Australia; 4 The Ottawa Hospital Cancer Centre, Ottawa, ON, Canada; 5 Leuven Cancer Institute, University Hospitals Leuven, Leuven, Belgium; 6 Mount Hospital, Perth, WA, Australia; 7 Wesley Medical Centre Brisbane, Auchenflower, QLD, Australia; 8 Department of OB/GYN, Medical University of Vienna, General Hospital, Vienna, Austria; 9 AstraZeneca, Wilmington, DE, USA; 10 AstraZeneca, Alderley Park, Macclesfield, UK; 11 Celgene, Seville, Spain

## Abstract

**Introduction:**

This Phase I study evaluated the safety, tolerability and efficacy of olaparib, a potent oral poly(ADP-ribose) polymerase (PARP) inhibitor, in combination with paclitaxel in patients with metastatic triple-negative breast cancer (mTNBC).

**Methods:**

Eligible patients who had received ≤1 prior cytotoxic regimen for mTNBC were treated with olaparib 200 mg bid continuously plus weekly paclitaxel 90 mg/m^2^ for three weeks per four-week cycle. Dose modifications in a large proportion of patients due to neutropenia resulted in enrollment of a second cohort of patients who, if they experienced grade ≥2 neutropenia in cycle 1, received granulocyte-colony stimulating factor, which was continued prophylactically in subsequent cycles. All patients had measurable disease; tumor responses were evaluated according to RECIST (version 1.0).

**Results:**

Nineteen patients (cohort 1, n = 9; cohort 2, n = 10) received treatment; 15 had received prior taxane chemotherapy. The most frequent adverse events were diarrhea (n = 12, 63%), nausea (n = 11, 58%) and neutropenia (n = 11, 58%). Seven neutropenia events were reported in cohort 1 (four grade ≥3) and four in cohort 2 (two grade ≥3, including one event of febrile neutropenia). The median (range) dose intensity of paclitaxel was 57% (26 to 100%) in cohort 1 and 73% (29 to 100%) in cohort 2. Seven patients (37%) had a confirmed partial response; one patient remains on olaparib monotherapy without progression.

**Conclusions:**

The combination of olaparib and weekly paclitaxel was complicated by a significant clinical interaction, with higher-than-expected rates of neutropenia despite secondary prophylaxis. Given the encouraging response rate, alternative scheduling and dosing strategies should be considered (funded by AstraZeneca; ClinicalTrials.gov, NCT00707707).

## Introduction

Triple-negative breast cancers (TNBC) are defined by the lack of expression of the estrogen receptor (ER), progesterone receptor (PR) and human epidermal growth factor receptor 2 (HER2) [1]. TNBC has distinct clinical and pathological characteristics and occurs at higher rates in younger women and in women of African-American descent [2,3]. Advanced TNBC confer an aggressive clinical course with a poor prognosis compared with other breast cancer subtypes. Most notably, patients who present with TNBC have a median survival of 7 to 13 months following recurrence, compared with greater than 20 months for patients with non-TNBC [4,5].

It is now recognized that TNBC is molecularly heterogeneous and there are ongoing efforts to define appropriate targets for directed therapy. With no confirmed single oncogenic driver, TNBC is not amenable to treatment with currently approved targeted approaches, such as trastuzumab or endocrine therapy, making chemotherapy treatment the main systemic treatment option. Evidence from studies of taxane-based chemotherapy regimens have indicated that patients with TNBC derive greater benefit from regimens that include a taxane than those that do not [6-8].

One of the first molecular insights into TNBC is the observation that a significant proportion of tumors arise in *BRCA1* mutation carriers and have gene expression profiles that are similar to those of BRCA-deficient tumors [9]. The *BRCA1* gene plays a critical role in DNA double-strand break repair, contributing to the maintenance of DNA stability. Poly(ADP-ribose) polymerase (PARP) enzymes, especially PARP-1, are critical for appropriate recognition and repair of DNA breaks. Tumor cell lines lacking functional BRCA1 or BRCA2 (most notably defects in homologous recombination) have been shown to be sensitive to PARP inhibitors in preclinical studies [10,11]. Demonstrating proof-of-principle, studies of olaparib, a potent oral PARP inhibitor, have demonstrated monotherapy activity and acceptable toxicity in patients with ovarian or breast cancer who have a germline *BRCA1* or *BRCA2* mutation [12,13]. However, in the subset of patients with a germline *BRCA1* or *BRCA2* mutation who participated in a Phase II study of olaparib monotherapy, no confirmed objective responses were observed in the eight patients with breast cancer [14].

Given the similarities between *BRCA1*-associated breast cancers and TNBC, it has been suggested that TNBC may be sensitive to therapeutic strategies that target DNA repair mechanisms.

In view of the preclinical and early clinical data reporting efficacy in tumors with homologous recombination defects, this study was initiated to evaluate the safety and tolerability of olaparib in combination with standard weekly paclitaxel in patients with metastatic TNBC (mTNBC).

## Methods

### Patients

Eligible female patients aged >18 years were enrolled at six centers in four countries. All patients were required to have histologically or cytologically, locally confirmed mTNBC (ER- and PR-negative [Allred score <3 or an IHC score of 0] and HER2-negative [IHC score of 0 or 1, or fluorescence *in situ* hybridization (FISH) negative] breast carcinoma); received ≤1 prior cytotoxic therapy regimen for metastatic disease; an Eastern Cooperative Group (ECOG) performance status ≤2; normal organ and bone marrow function; a minimum washout period of 12 months following any previous paclitaxel treatment; and a minimum washout period of 2 weeks following any other previous chemotherapy or radiotherapy.

All patients provided written informed consent. The study was approved by the independent ethics committee for each trial center: Melbourne Health Human Research Ethics Committee, Melbourne, Victoria, Australia; Bellberry Limited Human Research Ethics Committee, Ashford, South Australia, Australia; Mount Hospital Ethics Committee, Perth, Western Australia, Australia; Ethikkommission Medizinische Universität Wien, Vienna, Austria; Commissie Medische Ethiek van de Univ. Ziekenhuis K.U. Leuven, Leuven, Belgium; Ontario Cancer Research Ethics Board MARs Centre, Toronto, Ontario, Canada. The study was conducted in accordance with the Declaration of Helsinki [15], consistent with Good Clinical Practice and the AstraZeneca policy on bioethics [16].

### Study design

This was a Phase I, open-label, multicenter, safety study to establish the safety and tolerability of olaparib and paclitaxel (ClinicalTrials.gov identifier: NCT00707707). A double-blind, randomized Phase II portion of the study was planned if an acceptable dose was identified in Phase I.

Patients initially received olaparib 200 mg bid (4 × 50 mg capsules twice daily) in combination with paclitaxel 90 mg/m^2^ administered as an intravenous (iv) infusion over one hour on days 1, 8 and 15 of a 28-day cycle for 6 to 10 cycles (cohort 1). This dose of olaparib was chosen following the assessment of the pharmacokinetic (PK) and safety and tolerability profiles of olaparib in human monotherapy studies at doses of between 100 mg and 400 mg bid (the maximum tolerated dose) [12,13,17]. Toxicities were managed with olaparib and paclitaxel dose interruptions, and paclitaxel dose reductions to 65 mg/m^2^. After paclitaxel had been administered in combination with olaparib for 6 to 10 cycles at the discretion of the treating physician, paclitaxel was stopped and olaparib treatment was continued as 400 mg bid monotherapy until objective disease progression. Toxicities associated with olaparib monotherapy were managed by dose interruption; in the event of a toxicity recurring after dose interruption, or if olaparib dosing was interrupted owing to a grade ≥3 adverse event (AE), dose reduction to 200 mg bid (and then, if necessary, to 100 mg bid) was considered or required, respectively.

**Figure 1:**
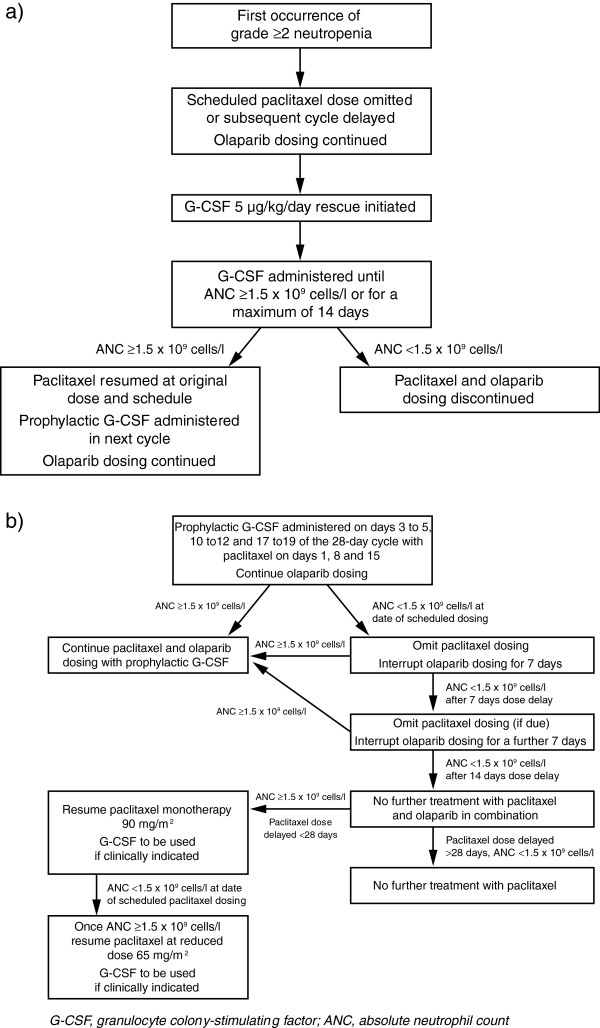
Management of (a) the first occurrence of neutropenia using rescue G-CSF and (b) subsequent treatment cycles in patients who have received rescue G-CSF in cohort 2.

A greater-than-expected occurrence of grade ≥2 neutropenia within the first two cycles of treatment resulted in paclitaxel dose modifications, including dose reductions and dosing delays. Consequently, a second cohort of patients was enrolled following a protocol amendment that included a stepwise approach to the management of neutropenia and allowed the use of prophylactic administration of granulocyte colony-stimulating factor (G-CSF) to enable optimal dose intensity of paclitaxel to be maintained. Patients in cohort 2 received olaparib and paclitaxel at the same doses and schedule as patients in cohort 1. For patients in cohort 2, upon first occurrence of grade ≥2 neutropenia, the paclitaxel dose was omitted or delayed; olaparib dosing was continued; and G-CSF 5 μg/kg/day was administered by subcutaneous (sc) injection until absolute neutrophil count (ANC) was ≥1.5 × 10^9^/l or for a maximum of 14 days (Figure 1a). Once ANC was ≥1.5 × 10^9^/l, then paclitaxel dosing was resumed at full dose and prophylactic G-CSF was administered in subsequent cycles. However, if ANC remained <1.5 × 10^9^/l after 14 days of treatment with G-CSF, then olaparib and paclitaxel were discontinued. Following the first occurrence of grade ≥2 neutropenia, prophylactic G-CSF 5 μg/kg/day sc was administered on days 3 to 5, 10 to 12 and 17 to 19 in subsequent cycles following paclitaxel dosing on days 1, 8 and 15 (Figure 1b). The management of subsequent treatment cycles in patients who received rescue G-CSF is depicted in Figure 1b. In cohort 1, G-CSF was prohibited during the first cycle of therapy, but permitted thereafter at the investigator’s discretion for management of neutropenia according to local hospital guidelines and local clinical practice. Prophylactic use of G-CSF was discouraged.

A minimum of six evaluable patients were required to complete two cycles of combination therapy. Therefore, it was expected that 10 patients per cohort would be required to ensure 6 evaluable patients.

### Study endpoints and assessments

The primary endpoint was evaluation of safety and tolerability of olaparib in combination with paclitaxel, assessed by the incidence and severity of AEs according to Common Terminology Criteria for Adverse Events (CTCAE) version 3.0. Secondary endpoints were evaluation of preliminary overall response rate (ORR) and progression-free survival (PFS) assessed by investigators according to Response Evaluation Criteria In Solid Tumors (RECIST) version 1.0. PFS was defined as the time from randomization to the earliest date of assessment of objective progression (per RECIST) or death by any cause in the absence of progression. Efficacy analyses were not originally planned for the Phase I part of the study, but these endpoints have been summarized as the study did not proceed into Phase II.

### Statistical analysis

Data are summarized descriptively as no formal statistical comparisons of the data were performed. The Wilson score method was used to calculate the 90% confidence intervals (CI), which are provided for preliminary ORR data as a measure of precision. Median PFS and 95% CIs were calculated using Kaplan–Meier methodology.

## Results

### Patients

Of 24 patients enrolled between 15 September 2008 and 21 April 2009, 19 received study treatment (n = 9, cohort 1; n = 10, cohort 2). Five patients were not assigned to treatment due to disease progression (n = 1), screening failure (n = 3) and voluntary withdrawal (n = 1). Baseline demographics are shown in Table 1; all patients were female and Caucasian. Five and six patients in cohorts 1 and 2, respectively, received at least six cycles of combination therapy and six patients in each cohort completed at least six cycles of olaparib treatment. Median actual olaparib treatment duration (excluding dose interruptions) was 168.0 days (range 50 to 389) in cohort 1 and 151.0 days (range 57 to 243) in cohort 2. Median dose intensity (total dose received/total dose planned) of olaparib was 100% (cohort 1 range 86 to 100%; cohort 2 range 80 to 100%) in both cohorts. Median dose intensity (total dose received/total dose planned) of paclitaxel was 57.2% (range 26 to 100%) in cohort 1 and 73.1% (range 29 to 100%) in cohort 2. At the time of data cut-off (9 November 2009), one patient in cohort 1 was ongoing (receiving olaparib only) and three patients in cohort 2 were ongoing (two were receiving olaparib plus paclitaxel and one olaparib only); one patient in cohort 2, who is not a *BRCA1/2* mutation carrier, was in complete radiological remission and still receiving olaparib in December 2012.

**Table 1:** Baseline demographics

	**Cohort 1**	**Cohort 2**	**Overall**
	**(n = 9)**	**(n = 10)**	**(n = 19)**
**Mean age, years (range)**	50.0 (36 to 71)	50.7 (38 to 67)	50.4 (36 to 71)
**ECOG status, n**			
**0/1/2**	8/1/0	5/5/0	13/6/0
**Previous chemotherapy regimens, n (%)**			
Any adjuvant regimen	5 (56)	9 (90)	14 (74)
Taxane^a^	7 (78)	8 (80)	15 (79)
First-line setting	2 (22)	0	2 (11)
Adjuvant setting	2 (22)	6 (60)	8 (42)
Neo-adjuvant setting	3 (33)	1 (10)	4 (21)
Metastatic setting	0	2 (20)	2 (11)
Anthracycline	6 (67)	6 (60)	12 (63)
Capecitabine	1 (11)	0	1 (5)

ECOG, Eastern Cooperative Group.

^a^One patient in cohort 2 had previously received taxane chemotherapy in both adjuvant and metastatic settings.

### Safety

All 19 patients experienced at least one AE. The majority of patients (16 (84%)) experienced at least one AE that was considered to be related to olaparib treatment. Thirteen patients (68%) had at least one CTCAE grade ≥3 event, and a greater proportion of these were in cohort 1 (89%) than cohort 2 (50%).

The most frequently reported CTCAE grade ≥3 events were neutropenia (44%) and anemia (22%) in cohort 1 and neutropenia (20%) in cohort 2 (Table 2). Seven (78%) and nine (90%) patients in cohorts 1 and 2, respectively, experienced AEs that were considered to be related to olaparib treatment, the most commonly reported were neutropenia (53%), diarrhea (42%), fatigue (37%) and nausea (26%). All 19 patients experienced AEs that were considered to be causally related to paclitaxel, the most common of which were neutropenia (58%), fatigue (53%), alopecia (53%) and diarrhea (47%). One patient died in cohort 1 (post follow-up) due to disease progression and multiple-organ failure.

**Table 2:** Adverse events reported by ≥30% of patients overall, by maximum reported grade

	**Evaluable patients, n (%)**				
	**Cohort 1**		**Cohort 2**		**Overall**
	**(n = 9)**		**(n = 10)**		**(n = 19)**
	**Grade 1/2**	**Grade ≥3**	**Grade 1/2**	**Grade ≥3**	
Diarrhea	6 (67)	0	6 (60)	0	12 (63)
Nausea	5 (56)	0	6 (60)	0	11 (58)
Neutropenia	3 (33)	4 (44)^a^	2 (20)	2 (20)^b^	11 (58)
Alopecia	6 (67)	0	4 (40)	0	10 (53)
Fatigue	6 (67)	0	3 (30)	1 (10)	10 (53)
Anemia	3 (33)	2 (22)	1 (10)	0	6 (32)
Constipation	4 (44)	0	2 (20)	0	6 (32)
Peripheral neuropathy	3 (33)	0	3 (30)	0	6 (32)
Rash	1 (11)	0	5 (50)	0	6 (32)
Vomiting	3 (33)	0	3 (30)	0	6 (32)

^a^Includes one grade 4 event.

^b^Includes one case of febrile neutropenia.

### Treatment dose modifications

Eight patients (89%) in cohort 1 had paclitaxel dose modifications (dose delay and/or dose reduction), six (67%) of whom had both a delay and a dose reduction. Four patients (44%) had olaparib dose modifications (dose reduction, n = 2; dose delay, n = 2) due to neutropenia; one of these patients also had a dose interruption of olaparib because of infection and anemia, and another of these four patients had a dose interruption of olaparib due to skin infection (of the breast) and skin disorder (skin breakdown).

In cohort 2, six patients (60%) had paclitaxel dose modifications, with three (30%) having both a delay and a dose reduction. Three patients (30%) had olaparib dose modifications; two patients (20%) due to neutropenia with one of these patients also undergoing dose modifications of olaparib due to pyrexia, herpes zoster and aphasia. The other patient had dose modifications of olaparib due to increased blood bilirubin, abnormal blood lactate dehydrogenase and abnormal gamma-glutamyltransferase.

### Efficacy

**Figure 2:**
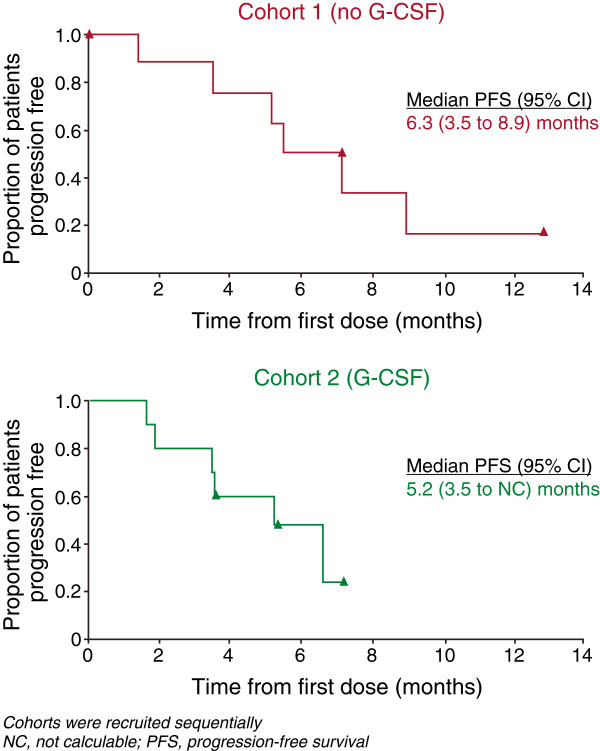
Kaplan–Meier curves of progression-free survival in cohorts 1 and 2.

ORR in cohort 1 was 3/9 patients (33.3%, 90% CI; 14.2 to 60.2%) and in cohort 2 was 4/10 patients (40%, 90% CI; 19.4 to 64.8%; Table 3). Median PFS (95% CI) was 6.3 (3.5 to 8.9) months for cohort 1 and 5.2 (3.5 - not calculable) months for cohort 2 (Figure 2). These estimated medians are based on very few events (six in each cohort) so should be interpreted with caution.

**Table 3:** Response rates according to RECIST

	**Evaluable patients, n (%)**	
**Responder status**	**Cohort 1**	**Cohort 2**
	**(n = 9)**	**(n = 10)**
**ORR**	3 (33)	4 (40)
**CR**	0	0^b^
**PR**^**a**^	3 (33)	4 (40)
**SD ≥7 weeks**	3 (33)	3 (30)
**Unconfirmed PR**^**a**^	1 (11)	2 (20)
**PD**	3 (33)	3 (30)

ORR, objective response rate; CR, complete response; PR, partial response; SD, stable disease; PD, progressive disease.

^a^One patient in cohort 1 and two patients in cohort 2 had unconfirmed partial responses and so were included in the SD seven or more weeks response category.

^b^Following data cut-off, one patient in cohort 2 achieved a CR and, as of December 2012, remained in complete radiological remission; no mutation in the *BRCA1* and/or *BRCA2* genes was identified in this patient by sequencing and multiplex ligation-dependent probe amplification (MLPA).

## Discussion

Current treatment options for TNBC are limited and there is no standard of care for patients with mTNBC who have progressed following treatment with standard chemotherapeutic regimens [18]. The use of PARP inhibitors to target DNA repair deficiencies in combination with systemic therapies continues to generate considerable clinical interest.

Previously, olaparib 400 mg bid monotherapy led to promising clinical outcomes in a proof-of-principle trial in patients with BRCA-deficient breast cancers, despite prior data suggesting that 100 mg bid might be sufficient to inhibit PARP [13,19]. We chose an olaparib dose of 200 mg bid for use in combination with standard paclitaxel dosing. In the Phase I run-in component of our study, which was performed to ensure safe delivery of olaparib with paclitaxel, the combination had a generally manageable toxicity profile in patients with TNBC. However, despite this patient cohort not being heavily pre-treated and not having excessive bone marrow involvement, the treatment combination was associated with a greater-than-expected incidence and severity of neutropenia, which resulted in delivery of a lower paclitaxel dose intensity than planned. Although the administration of prophylactic G-CSF to patients in cohort 2 led to an increase in paclitaxel dose intensity (median intensity 73% vs 57% in cohort 1), the paclitaxel dose was still lower than anticipated and the rate of neutropenia remained high (20%). A suitable dosing schedule could not be identified for the Phase II part of our study; therefore, the study was terminated at the end of Phase I.

Historically, single-agent paclitaxel in the metastatic setting has demonstrated much lower rates of grade 3/4 neutropenia (from 0.3% as a first-line treatment to 15% in heavily pre-treated patients) than observed in combination with olaparib in our study [20,21]. Dose-limiting toxicities of myelosuppression have been noted in studies of other PARP inhibitors in combination with chemotherapy agents [22-26]. In addition, thrombocytopenia and neutropenia were the most common grade ≥3 toxicities in a Phase II trial of the PARP inhibitor veliparib, in combination with the oral alkylating agent temozolomide in patients with metastatic breast cancer [27]. Olaparib has previously been evaluated in combination with chemotherapy in patients with *BRCA1/2*-mutant cancer or sporadic cancer; the majority of these trials involved treatment combinations that were expected to be synergistic due to their effects on DNA repair and hence potentiate myelotoxicity. In a Phase I dose-finding study of olaparib in combination with carboplatin, the initial continuous schedule of olaparib dosing was changed to intermittent administration because of thrombocytopenia and delayed recovery of neutropenia [28]. However, the combination of olaparib 200 mg bid with carboplatin and paclitaxel was recently shown to have an acceptable tolerability profile in a Phase II trial in patients with serous ovarian cancer [29]. The current study evaluated the combination of olaparib and paclitaxel, which was not expected to potentiate myelotoxicity. Potential confounders to explain the toxicity profile experienced by patients in our study include pharmacodynamic and PK interactions, such as the timing and sequencing of chemotherapy with olaparib or off-target effects through inhibition of tankyrases [30]. It is not yet clear whether neutropenia is potentially a surrogate for clinical activity. The combination of olaparib with paclitaxel is currently being evaluated in patients with gastric cancer (ClinicalTrials.gov identifier: NCT01063517) and advanced solid tumors (ClinicalTrials.gov identifier: NCT00516724).

Despite the decreased paclitaxel dose intensity in our study, encouraging response rates were observed following treatment with olaparib plus paclitaxel with 3/9 patients (33%) and 4/10 patients (40%) in cohorts 1 and 2, respectively, achieving partial responses. Our response rates are higher than those reported in both a Phase II study of paclitaxel monotherapy in women with metastatic breast cancer (21.5%; n = 38/177) and a recent study of olaparib monotherapy, in which no confirmed responses were observed among the 23 evaluable patients with advanced metastatic or recurrent breast cancer [14,21]. The response rates in our study are also notable given the heterogeneous nature of TNBC and the limited treatment options for this disease. From this small study, the subtypes of patients with TNBC who are most likely to respond to olaparib treatment could not be evaluated; however, responses to olaparib have been seen in previous studies of patients with breast and ovarian cancers with germline *BRCA1* and/or *BRCA2* mutations, as well as patients with high-grade serous ovarian cancers, suggesting that there is a subgroup who will be likely to benefit [12-14]. Future studies should assess the mutation status of each patient to further identify those who are most likely to respond to this treatment strategy. In addition, future studies should incorporate other molecular measures of functional homologous recombination deficiency for their sensitivity to PARP inhibition [31].

## Conclusion

In summary, although the incidence of neutropenia observed in our study was higher than would be expected with either olaparib or paclitaxel alone, the combination of olaparib and paclitaxel had a generally manageable toxicity profile and preliminary evidence of antitumor activity was observed. The optimal schedule of olaparib administration in combination with paclitaxel was not defined in this study, leading to its early termination. The ongoing trials of olaparib in combination with paclitaxel should help to identify a suitable treatment schedule for this combination. Currently, it remains unclear whether the best use of PARP inhibitors will be in combination with another type of DNA-damaging agent, or with a standard cytotoxic chemotherapy agent to achieve optimal benefit in patients with TNBC. Therefore, further studies investigating the safety and efficacy of olaparib in combination with DNA-damaging agents, cytotoxic chemotherapy and as a monotherapy are indicated in this setting.

## Abbreviations

AE: Adverse event; ANC: Absolute neutrophil count; BRCA1/2: Breast cancer 1/2; CI: Confidence interval; CR: Complete response; CTCAE: Common terminology criteria for adverse events; DNA: Deoxyribonucleic acid; ECOG: Eastern cooperative group; ER: Estrogen receptor; FISH: Fluorescence *in situ* hybridization; G-CSF: Granulocyte colony-stimulating factor; HER2: Human epidermal growth factor receptor 2; IHC: Immunohistochemistry; iv: Intravenous; MLPA: Multiplex ligation-dependent probe amplification; mTNBC: Metastatic triple-negative breast cancer; ORR: Overall response rate; PARP: Poly(ADP-ribose) polymerase; PD: Progressive disease; PFS: Progression-free survival; PK: Pharmacokinetic; PR: Progesterone receptor; PR: Partial response (Table 3 only); RECIST: Response evaluation criteria in solid tumors; sc: Subcutaneous; SD: Stable disease; TNBC: Triple-negative breast cancer.

## Competing interests

RD, GL, HW, AC, NM and CS have no conflicts of interest to disclose. MC has received remuneration from AstraZeneca for a consultant/advisory role. EL and CW are employees of AstraZeneca and own stock. JC is a previous employee of AstraZeneca and owns stock.

## Authors’ contributions

RD, MC, EL and JC were involved in the conception and design of the manuscript. RD, GL, AC, CS, EL and JC contributed to the collection and assembly of data. RD, GL, HW, AC, NM, CS, EL, CW and JC contributed to the analysis and interpretation of the data. JC provided administrative support. RD, HW, AC and CS provided study material/patients. All the authors were involved in writing the draft manuscript and approved the final manuscript.
